# Case Report: Posterior reversible encephalopathy syndrome after lenvatinib treatment for hepatocellular carcinoma

**DOI:** 10.3389/fphar.2025.1487009

**Published:** 2025-03-21

**Authors:** Minchun Chen, Jing Shen, Rongrong Jia, Mingze Chang, Jingyi Zhang, Jie Zheng, Runqing Xue, Lulu Guo, Kangkang Yan

**Affiliations:** ^1^ Department of Pharmacy, Xi’an No.3 Hospital, The Affiliated Hospital of Northwest University, Xi’an, China; ^2^ Department of Neurology, Xi’an No.3 Hospital, The Affiliated Hospital of Northwest University, Xi’an, China; ^3^ Department of Medical Imaging, Xi’an No.3 Hospital, The Affiliated Hospital of Northwest University, Xi’an, China

**Keywords:** hepatocellular carcinoma, posterior reversible encephalopathy syndrome, lenvatinib, adverse drug reactions, PRES

## Abstract

**Background:**

Posterior reversible encephalopathy syndrome (PRES) is characterized by headaches, vision loss, confusion, encephalopathy, seizures, and reversible focal edema on neuroimaging. Early recognition and treatment of PRES are essential to prevent severe complications. Lenvatinib is a multi-targeted kinase inhibitor that is used as a first-line treatment for patients with hepatocellular carcinoma (HCC). Lenvatinib-induced PRES is a less commonly recognized side effect.

**Case presentation:**

A 72-year-old female patient with HCC, who had no history of hypertension, received lenvatinib therapy. The patient exhibited symptoms such as confusion, altered mental status, headaches, and severe hypertension during treatment. Neuroimaging revealed characteristic findings of vasogenic edema in the white matter of the brain. The patient’s neurological symptoms gradually improved after lenvatinib discontinuation, and follow-up imaging showed a reduction in the white matter abnormalities.

**Conclusion:**

The underlying mechanisms of PRES induced by lenvatinib remain unclear, but hypertension is considered a crucial factor in its pathogenesis. This case report adds to the understanding of the potential adverse effects associated with lenvatinib in patients with HCC, emphasizing the need for vigilance in monitoring and managing such complications to ensure the safety and wellbeing of patients undergoing this treatment.

## Introduction

Hepatocellular carcinoma (HCC) is a life-threatening cancer that has emerged as a global health concern (Sung et al., 2021). In 2020, primary liver cancer ranked among the top three causes of cancer-related deaths, leading to 830,000 deaths, and was the sixth most commonly diagnosed cancer with approximately 906,000 new cases. HCC accounted for approximately 75%–85% of all newly diagnosed primary liver cancer cases (Sung et al., 2021). If detected early, HCC can be surgically treated, ablated, or transplanted. However, systemic therapy may be required in approximately 50%–60% of patients with HCC (Vogel et al., 2022). Over the past few decades, significant progress has been made in the systemic treatment of HCC. Currently, the most common effective strategies for HCC treatment are multi-kinase inhibitors and immune checkpoint inhibitors (Sankar et al., 2024). According to the current Chinese guidelines, lenvatinib is the standard first-line treatment strategy for HCC (Xie et al., 2023).

Lenvatinib, an oral multi-targeted kinase inhibitor, targets vascular endothelial growth factor receptors (VEGFR) 1-3, fibroblast growth factor receptors 1-4, platelet-derived growth factor receptors α, and rearranged during transfection and stem cell factor receptor proto-oncogenes (Suyama and Iwase, 2018). The phase III REFLECT trial showed that lenvatinib was non-inferior to sorafenib in terms of overall survival and established it as a preferred treatment option (Kudo et al., 2018). In this trial, adverse events (AEs) such as hypertension, diarrhea, decreased appetite, and decreased weight were most frequently associated with lenvatinib treatment (Kudo et al., 2018). Similar findings have been observed in several studies (Ikeda et al., 2017; Hatanaka et al., 2020; Ohki et al., 2020; Shimose et al., 2020). However, few reports on neurotoxicity have been published. Headaches are among the most common AEs associated with lenvatinib treatment for progressive, radioiodine-refractory differentiated thyroid cancer (Nair et al., 2015). In addition, some rare AEs, including consciousness disorder and hypersomnia, were reported. In the literature, only a few cases of posterior reversible encephalopathy syndrome (PRES) related to lenvatinib have been reported (Matsuura et al., 2022; Tseng et al., 2022; Osawa et al., 2018; Abhishek et al., 2022), and PRES has not been previously reported in patients with HCC treated with lenvatinib.

In this study, a case of HCC with lenvatinib-induced neurological manifestations of PRES is presented.

## Case description

In 2018, a 72-year-old woman was diagnosed with T2N0M0 HCC (BCLC stage B, *Child-Pugh* A). Her medical and surgical history included 4-year coronary heart disease, 10-year paroxysmal atrial fibrillation, and resection of intestinal tuberculosis obstruction 33 years ago. It is crucial to note that our patient had no history of hypertension or diabetes mellitus. The patient has received treatment with traditional Chinese medicine and has declined surgery, transarterial chemoembolization (TACE) for early-stage HCC, and biopsy for the qualitative detection of liver tumors. A follow-up computer tomography (CT) reexamination in 2019 revealed a reduction in the lesion.

**FIGURE 1:**
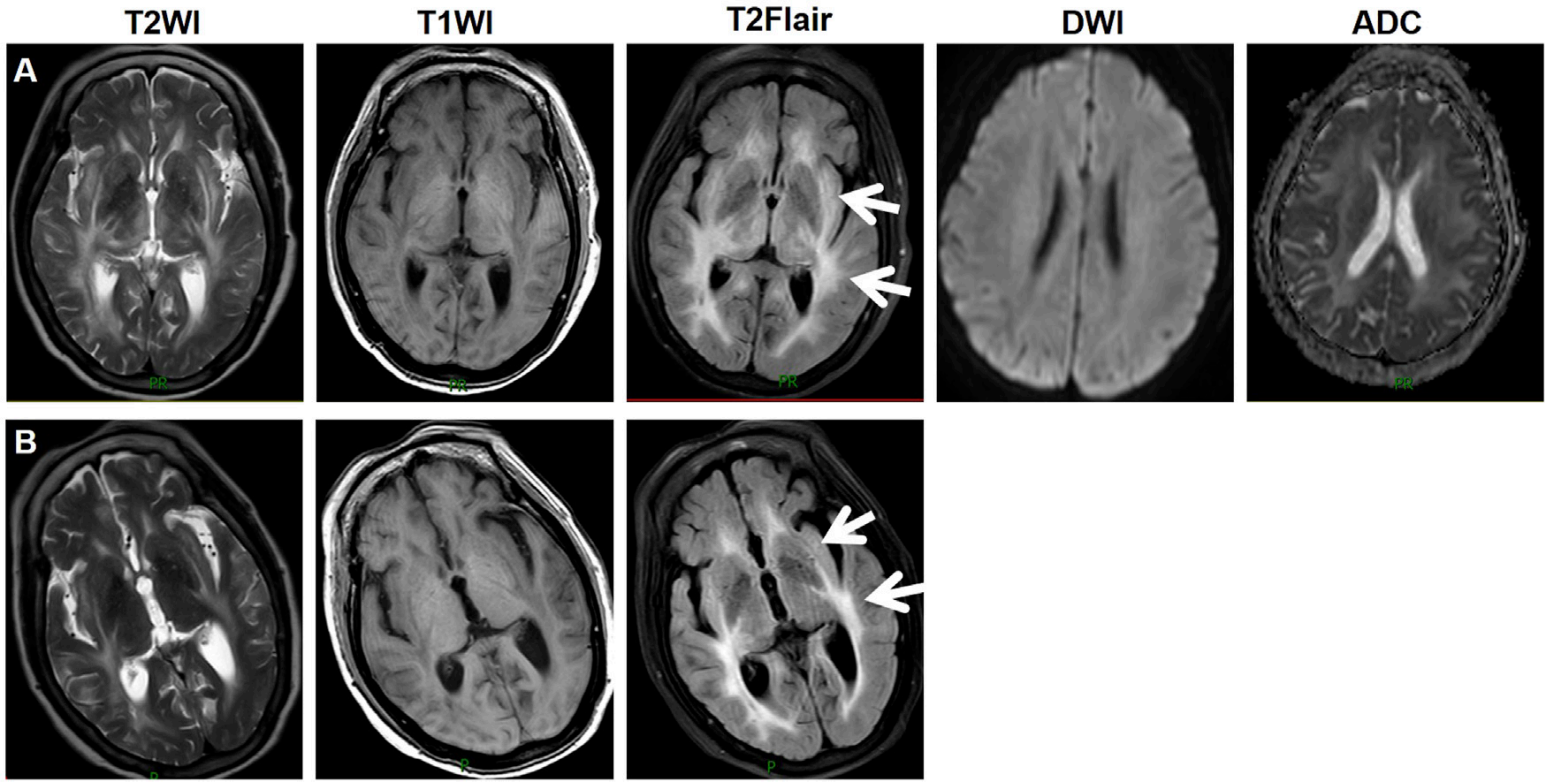
Imaging manifestations of the patient. On August 30, an MRI revealed bilaterally distributed hyperintensity of the white matter in the periventricular and basal ganglia on T2 images, and fluid-attenuated inversion recovery (FLAIR) images. Restricted diffusion was not observed on diffusion-weighted imaging or the apparent diffusion coefficient **(A)**. On September 4, a follow-up MRI reexamination indicated a slight reduction in the signal abnormality in the bilateral white matter **(B)**. Abbreviations: T2WI, T2 weighted imaging; T1WI, T1 weighted imaging; T2Flair, T2 fluid attenuated inversion recovery; DWI, diffusion-weighted imaging; ADC, apparent diffusion coefficient.

During treatment, CT and magnetic resonance imaging (MRI) identified a hepatic metastatic tumor on 15 May 2023, indicating disease progression. However, the patient chose not to undergo TACE, and on 1 July 2023, lenvatinib 8 mg QD was initiated for salvage therapy. Initially, the patient did not experience any adverse reactions. Lenvatinib therapy was continued due to unresectable HCC. She had been well until 1 week prior when she started experiencing confusion, difficulty identifying familiar people, and a longer sleep duration. Three days ago, the patient experienced a consciousness disorder, including progressive deterioration of consciousness and double incontinence. Furthermore, she had a blood pressure (BP) of 204/120 mmHg, despite having no history of hypertension, and suddenly developed a severe headache. The patient was admitted on 29 August 2023, due to abnormal mental behavior. During the neurological examination, the patient entered a state of lethargy. Furthermore, the patient had muscle strength and normal symmetrical reflexes. CT, MRI, and magnetic resonance angiography images revealed no signs of intracranial hemorrhage or infarction. Biochemical testing, including complete blood cell count, blood chemistry, coagulation, and kidney function tests did not detect any significant abnormalities, with an estimated glomerular filtration rate (eGFR) of 99.186 mL/min. The patient exhibited mild hepatic dysfunction characterized by predominantly unconjugated hyperbilirubinemia (total bilirubin: 34.8 μmol/L; direct bilirubin: 5.6 μmol/L; indirect bilirubin: 24.9 μmol/L). Notably, hepatocellular integrity was preserved, as evidenced by normal transaminase levels (aspartate aminotransferase: 28 U/L; alanine aminotransferase: 32 U/L) with normal serum ammonia levels (23 μmol/L). Immunological evaluation revealed weakly positive autoimmune markers (anti-histone antibody: 1:50; antinuclear antibody: 1:100, speckled pattern), while comprehensive serological testing excluded antiphospholipid syndrome (negative for lupus anticoagulant, anti-cardiolipin, and anti-β2-glycoprotein I antibodies) and systemic vasculitis (negative for anti-neutrophil cytoplasmic antibodies and anti-endothelial cell antibodies). On August 30, an MRI revealed bilaterally distributed hyperintensity of the white matter in the periventricular and basal ganglia on T2 images, and fluid-attenuated inversion recovery (FLAIR) images. However, restricted diffusion was not observed on diffusion-weighted imaging or the apparent diffusion coefficient (Figure 1A). These observations indicated vasogenic edema, leading to suspicion of Fazekas grade 3 PRES. Cerebrospinal fluid (CSF) analysis revealed normal glucose levels and cell counts, along with increased albumin (2432 mg/L, normal range: 150–450 mg/L). Comprehensive relevant diagnostic testing, including tests for metabolic, demyelinating, vascular, and tumoral conditions, ruled out any relevant issue. Drug toxicity could not be excluded due to progressive encephalopathy. Therefore, lenvatinib was immediately discontinued, and BP was controlled using intravenous urapidil at 130–140/80–90 mmHg. After 5 days, her neurological symptoms gradually improved. On September 4, a follow-up MRI reexamination indicated a slight reduction in the signal abnormality in the bilateral white matter (Figure 1B). The patient was discharged after 7 days of admission.

## Discussion

PRES is a neurological syndrome characterized by headache, seizures, visual disturbances, or altered mental status, along with bilateral posterior white and/or gray matter lesions. The primary radiological manifestation is white matter vasogenic edema in the posterior cortex (Hinchey et al., 1996). As implied by their name, most PRES cases can be reversed. A key point is the prompt recognition and early diagnosis of PRES and the addressing of the underlying cause (Fugate and Rabinstein, 2015). The treatment of PRES involves providing support, controlling BP, stopping seizures, inhibiting brain edema, and withholding drugs and toxic agents that induce PRES (Geocadin, 2023; Triplett et al., 2022). The patients recovered from neurological deficits and radiological films within 2 weeks. However, imaging studies indicate that poor outcomes and death are associated with cerebral hemorrhage and cerebral infarction (Geocadin, 2023).

Many drugs can cause PRES (Geocadin, 2023; Triplett et al., 2022; Balcerac et al., 2023). In recent years, numerous new anticancer molecules have been introduced. A correlation between PRES and cancer has been observed in patients who use many targeted agents, particularly antiangiogenic agents, including pazopanib (Deguchi et al., 2018), bevacizumab (Sclafani et al., 2012), and sorafenib (Laruelle et al., 2018). In phase III REFLECT trial, no patients who received lenvatinib 8 or 12 mg orally once daily for HCC developed PRES. However, in the SELECT trial, 0.3% of patients with differentiated thyroid cancer (DTC) who were orally administered lenvatinib at a daily dose of 24 mg developed PRES (FDA Label, 2021). Consequently, we presented the first case of PRES in a patient treated with lenvatinib for HCC.

A study demonstrated that the vasogenic edema caused by severe hypertension could trigger PRES (Xie and Jones, 2016). Hypertension was observed in 73% of patients in SELECT (DTC) and 45% of patients in REFLECT (HCC) (FDA Label, 2021). Notably, lenvatinib inhibits VEGFR, and VEGF is a key regulator of angiogenesis in tumors. Blocking VEGF/VEGFR signaling significantly inhibits tumors invasion and metastasis (Suyama and Iwase, 2018). VEGF regulates vasomotor tone and maintains BP by promoting nitric oxide production (Ancker et al., 2017). Hypertension of grades 2 or 3 developed in this patient during treatment despite having no history of hypertension. Therefore, lenvatinib-induced hypertension may be attributed to the appearance of PRES. In addition, a slow recovery from hypertension was observed after lenvatinib was discontinued. According to the National Cancer Institute guidelines, all patients should have a target BP of 140/90 mmHg or less during treatment with VEGFR inhibitors. It is also worth noting that anti-hypertensive drugs and BP monitoring may prevent potential PRES in patients receiving lenvatinib treatment.

The mechanism by which antiangiogenic drugs induce PRES remains unclear. Hypertension plays a vital role in the pathogenesis of PRES and appears to be associated with acute, severe hypertension that causes damage to the blood–brain barrier (BBB), leading to focal vasogenic edema (Geocadin, 2023). In addition, an increasing body of evidence indicates that endothelial dysfunction is a significant factor in this process (Marra et al., 2014). Lenvatinib, as a VEGF inhibitor, is closely associated with endothelial dysfunction, resulting in damage to the BBB and the development of vasogenic edema. In this case, abnormal lesions were observed in the white matter of the bilateral cerebrum with high T2-weighted signal intensity, high Flair-weighted signal intensity, and low T1-weighted signal intensity, indicating vasogenic edema. Therefore, lenvatinib-induced hypertension and vascular endothelial cell injury in patients without a history of hypertension may have played a role in PRES. After 7 days of interruption, the patient experienced remission of symptoms and typical imaging features. Lenvatinib is primarily metabolized by the hepatic enzymes CYP3A4. As the patient is not taking strong CYP3A4 modulators, the risk of pharmacokinetic interactions is considered low. For HCC patients, while a daily dose of 12 mg of lenvatinib has demonstrated promising anti-cancer efficacy, pharmacokinetic modeling suggests that this dose may lead to an excessively high area under the curve, increasing the risk of vascular toxicity (Hussein et al., 2017). Therefore, it is recommended that HCC patients weighing less than 60 kg receive 8 mg of lenvatinib daily, while those weighing 60 kg or more should receive 12 mg daily (Tamai et al., 2017). Given the patient’s individual characteristics—a 78-year-old male weighing 60 kg, with Child-Pugh A liver cirrhosis and an eGFR of 99.186 mL/min—a daily dose of 8 mg is selected to optimize the risk-benefit ratio. The causal relationship between a suspected drug and the adverse event was investigated using the Naranjo algorithm indicating an association between the syndrome and lenvatinib treatment.

Our analysis systematically compared the PRES characteristics associated with various antiangiogenic agents—including pazopanib, bevacizumab, and sorafenib—with the current case (Deguchi et al., 2018; Sclafani et al., 2012; Laruelle et al., 2018). Three patients had no prior history of systemic hypertension, while one had well-controlled hypertension. All cases developed new-onset or exacerbated hypertension accompanied by neurological symptoms 2–5 weeks after initiating treatment. MRI revealed classic posterior-predominant vasogenic edema involving the parieto-occipital lobes. Immediate discontinuation of anti-angiogenic agents, along with intensifying antihypertensive treatment was implemented. Neurological symptoms resolved within 5–9 days following treatment cessation, and MRI-confirmed edema resolution was noted at the 2-week follow-up. Two patients resumed anti-angiogenic therapy under stringent BP monitoring and prophylactic antihypertensive regimens, with no recurrence of PRES. These cases highlight the crucial interaction between anti-angiogenic pharmacology and hemodynamic stress in the pathogenesis of PRES. Proactive BP management and structured rechallenge protocols can mitigate risks without compromising oncologic efficacy.

The patient developed marked CSF hyperproteinorrhachia following lenvatinib initiation, indicative of BBB, a hallmark of PRES. Neuroimaging revealed characteristic periventricular white matter edema on MRI, aligning with PRES diagnostic criteria. Mechanistically, lenvatinib’s antiangiogenic effects likely compromise vascular endothelial integrity, increasing BBB permeability (Suyama and Iwase, 2018). This vascular dysfunction promotes the extravasation of plasma proteins, including albumin, into the CSF, leading to the observed CSF hyperproteinorrhachia. Notably, quantitative analysis reveals a significant positive correlation between vasogenic edema volume and CSF albumin concentration (r = 0.48,p < 0.001) (Neeb et al., 2016). Concomitantly, lenvatinib-induced hypertension likely synergized with endothelial injury, creating a vicious cycle that exacerbated BBB breakdown (Kudo et al., 2018). This dual pathogenesis–pharmacodynamic vascular toxicity compounded by hemodynamic stress–underscores the need for vigilant monitoring of neurological symptoms and blood pressure control during tyrosine kinase inhibitor (TKI) therapy.

The difference between PRES and hepatic encephalopathy (HE) is key to distinguishing lenvatinib-related adverse effects. Both conditions can manifest with altered mental status as seen in this patient. Additionally, both PRES and HE may lead to changes in brain imaging, particularly within the white matter. PRES typically presents with bilateral hyperintensities in the white matter, often affecting the occipital and parietal lobes (Geocadin, 2023). In contrast, HE may show white matter changes but more commonly involves the basal ganglia and frontal regions (Zhang and Zhang, 2018). In this case, the MRI revealed bilateral hyperintensity in the periventricular white matter, a hallmark feature of PRES. The pathophysiology of PRES is primarily related to endothelial dysfunction and BBB disruption, which can be caused by factors like severe hypertension, preeclampsia, or the use of antiangiogenic drugs such as lenvatinib (Geocadin, 2023). Given the patient’s hypertension and use of lenvatinib, these factors likely contributed to the development of PRES. On the other hand, HE is a consequence of liver dysfunction, which leads to the accumulation of toxic metabolites, particularly ammonia, that disrupt brain function (Butterworth, 2016). The patient’s liver cancer and potential liver failure might suggest HE; however, the patient’s normal serum ammonia level and the significant increase in CSF albumin further support a diagnosis of PRES.

This case provides the first documented association between lenvatinib and PRES in HCC patients, but several limitations must be considered. As a single-case observation, causality and incidence cannot be established, and the lack of comparative data from larger cohort limits generalizability. Systematic pharmacovigilance analyses of TKI-related neurotoxicity registries (e.g., WHO or FAERS) could identify additional cases and quantify reporting odds ratios. Prospective studies with serial neuroimaging and CSF biomarker monitoring in high-risk patients—especially those with uncontrolled hypertension or pre-existing microvascular disease—could clarify the temporal relationship between lenvatinib exposure and BBB dysfunction. Clinicians should remain vigilant for PRES in HCC patients receiving lenvatinib, particularly during early treatment phases or dose escalation.

Although the patient demonstrated significant clinical and radiological improvement following the discontinuation of lenvatinib and the initiation of antihypertensive therapy, the limited follow-up period restricts our ability to comprehensively evaluate potential long-term neurological sequelae or the recurrence of PRES. Notably, persistent microstructural brain changes have been observed in 10%–20% of PRES survivors, despite apparent clinical recovery (Frick et al., 2017). Furthermore, the reintroduction of therapies in cancer patients with a prior history of PRES has been associated with symptom recurrence in up to 8% of cases, underscoring the need for prolonged monitoring in this patient population (Gewirtz et al., 2021). In clinical practice, we recommend neurological follow-ups at least every 6 months for these patients, alongside a careful reassessment of rechallenge strategies, in collaboration with multidisciplinary oncology teams.

## Conclusion

To the best of our knowledge, this is the first case report of lenvatinib-induced PRES for HCC, and symptoms typically disappear within a few days to weeks after discontinuation of medications. The underlying mechanisms of PRES induced by lenvatinib remain unclear, but hypertension is considered a crucial factor in its pathogenesis. Management of PRES typically involves discontinuation of the causative drug, control of hypertension, and supportive care. Early recognition, diagnosis, and treatment of PRES are crucial for preventing serious complications.

## Data Availability

The original contributions presented in the study are included in the article/supplementary material, further inquiries can be directed to the corresponding author.
